# Sc-GPE: A Graph Partitioning-Based Cluster Ensemble Method for Single-Cell

**DOI:** 10.3389/fgene.2020.604790

**Published:** 2020-12-15

**Authors:** Xiaoshu Zhu, Jian Li, Hong-Dong Li, Miao Xie, Jianxin Wang

**Affiliations:** ^1^School of Computer Science and Engineering, Yulin Normal University, Yulin, China; ^2^Hunan Provincial Key Laboratory on Bioinformatics, School of Computer Science and Engineering, Central South University, Changsha, China

**Keywords:** single-cell clustering, cluster ensemble, consensus matrix, importance score, graph partitioning

## Abstract

Clustering is an efficient way to analyze single-cell RNA sequencing data. It is commonly used to identify cell types, which can help in understanding cell differentiation processes. However, different clustering results can be obtained from different single-cell clustering methods, sometimes including conflicting conclusions, and biologists will often fail to get the right clustering results and interpret the biological significance. The cluster ensemble strategy can be an effective solution for the problem. As the graph partitioning-based clustering methods are good at clustering single-cell, we developed Sc-GPE, a novel cluster ensemble method combining five single-cell graph partitioning-based clustering methods. The five methods are SNN-cliq, PhenoGraph, SC3, SSNN-Louvain, and MPGS-Louvain. In Sc-GPE, a consensus matrix is constructed based on the five clustering solutions by calculating the probability that the cell pairs are divided into the same cluster. It solved the problem in the hypergraph-based ensemble approach, including the different cluster labels that were assigned in the individual clustering method, and it was difficult to find the corresponding cluster labels across all methods. Then, to distinguish the different importance of each method in a clustering ensemble, a weighted consensus matrix was constructed by designing an importance score strategy. Finally, hierarchical clustering was performed on the weighted consensus matrix to cluster cells. To evaluate the performance, we compared Sc-GPE with the individual clustering methods and the state-of-the-art SAME-clustering on 12 single-cell RNA-seq datasets. The results show that Sc-GPE obtained the best average performance, and achieved the highest NMI and ARI value in five datasets.

## Introduction

Single-cell RNA sequencing (scRNA-seq) data measures the gene expression level in individual cells instead of the average gene expression level in bulk RNA-seq cells (Stuart and Satija, 2019). So, it has advantages in accurately identifying the transcriptomic signatures for cell types (Grün et al., 2015). Along with the rapid development of scRNA-seq technologies, the cost of sequencing is reduced, and larger datasets are generated, carrying a higher error rate (Vitak et al., 2017). The development brought some computational challenges (Kiselev et al., 2019; Zhu et al., 2019a), for example, (1) high noise. The drop-out rate from reverse transcription failure and sequencing depth would reach 80% (Soneson and Robinson, 2018; Andrews and Hemberg, 2019); (2) high dimension. The dimension usually exceeds 10,000, making it difficult to measure the similarity of cell pairs; (3) larger sample size. The sample size increases from dozens to hundreds of thousands, which raises the time and complexity involved in identifying cell types (Grun, 2020).

Clustering is an efficient way of analyzing scRNA-seq data to identify novel cell types, and some single-cell clustering methods are proposed (Xu et al., 2019; Yip et al., 2019). However, it can be observed that the clustering results from various clustering methods are different in the number of clusters and cell assignments. Meanwhile, no method performs best on all scRNA-seq datasets. The reason is that the existing methods focus on a different step in identifying cell types, including data denoising (Wang et al., 2018), dimensionality reduction (Wang and Gu, 2018; Becht et al., 2019), similarity measurement (Kim et al., 2019) and clustering (Qi et al., 2019; Zhu et al., 2019b). Notably, the similarity measurement plays an important role in identifying cell types. Some graph partitioning-based clustering methods achieved better performance for the accurate similarity measurement. For example, SNN-cliq (Xu and Su, 2015) constructed a weighted shared nearest neighbor (SNN) graph; and clustered cells by partitioning the cliques on the graph. PhenoGraph (Levine et al., 2015) performed another weighted strategy to generate an SNN graph; and partitioned the graph using the Louvain community detection method. SSNN-Louvain (Zhu et al., 2020) integrated the structural information to construct a structural SNN graph; and clustered cells by modifying the Louvain community detection method. The cells are sorted as per their importance in the initialization step of Louvain community detection method. MPGS-Louvain (Zhu et al., 2019c) constructed a novel global and path-based similarity graph, and also partitioned it using a modified Louvain community detection method. Therefore, it is a challenge to enhance the accuracy of clustering by combining more efficient clustering information in multiple views.

An increasing number of research shows that the cluster ensemble method is a good idea, which integrates the information of each clustering method in a different view (Kuncheva and Vetrov, 2006; Vega-Pons and Ruiz-Shulcloper, 2011; Liu et al., 2019). ISSCE (Yu et al., 2016) designed a clustering ensemble strategy to cluster high dimensional data, including three steps: firstly, the incremental approach was implemented to select clustering members; secondly, the random subspace division was applied to handle high dimensional data; finally, the constraint propagation method was used to integrate prior knowledge. Recently, some cluster ensemble methods for scRNA-seq data have been proposed. SC3 (Kiselev et al., 2017) ensembled several clustering results from *k*-means algorithm into a consensus matrix; and clustered cells using hierarchical clustering (HC). SAFE-clustering (Yang et al., 2019) implemented a hypergraph-based strategy to ensemble CIDR, Seurat, tSNE, and SC3 to construct a consensus matrix. *k*-means was used to cluster cells. They also proposed the SAME-clustering (Huh et al., 2020) methods by using a consensus matrix-based strategy to ensemble the same four clustering methods and combining the Expectation-Maximization algorithm to cluster cells. We find that these cluster ensemble methods are based on hypergraph-based or voting-based integrated learning and do not consider the different importance of the individual clustering method.

According to the principle that the minority is subordinate to the majority, we assume that the more consistent the cluster labels predicted by different clustering methods are, the more accurate they will be. That is, the individual clustering method with a higher similarity to others would be more important in the cluster ensemble strategy. Base on this assumption, we propose a novel graph partitioning-based ensemble method for single-cell clustering (Sc-GPE), integrating SNN-cliq, PhenoGraph, SSNN-Louvain, MPGS-Louvain, and SC3 by a weighted voting-based method. To measure the importance of the individual clustering method, we design a scoring strategy based on the adjusted rand index (ARI) (Hubert and Arabie, 1985). Then we construct a weighted consensus matrix, the weight is a score of the importance of each method. Finally, HC is performed to cluster cells. To prove the performance, Sc-GPE is compared to the five original clustering methods and the state-of-the-art cluster ensemble method “SAME-clustering.” The results demonstrate that Sc-GPE outperforms other methods.

## Materials and Methods

According to the analysis above, we can find that integrating multiple clustering results would merge more information in different views. Moreover, different clustering methods play different roles in integration. Inspired by these ideas, we propose the Sc-GPE method by ensembling five graph partitioning-based clustering methods which are SNN-cliq, PhenoGraph, SSNN-Louvan, MPGS-Louvain, and SC3. The main reasons for choosing the five clustering methods are as follows: firstly, the first four clustering methods are graph partitioning-based methods, and the last one is the consensus matrix-based method. Their good performance provides the basis to improve the accuracy of the cluster ensemble. Secondly, in the five clustering methods, different strategies of similarity graph construction and graph partitioning have been implemented, respectively. They would enhance the generalization ability of clustering. Sc-GPE has three following advantages: (1) it does not need to deal with the problem of different cluster labels from different cluster methods, so it is suitable for unsupervised clustering lacking the true cluster labels; (2) It is easy to implement since no special parameters need to be adjusted; (3) The weighted strategy is comprehensible and effective.

### Sc-GPE

**Figure 1:**
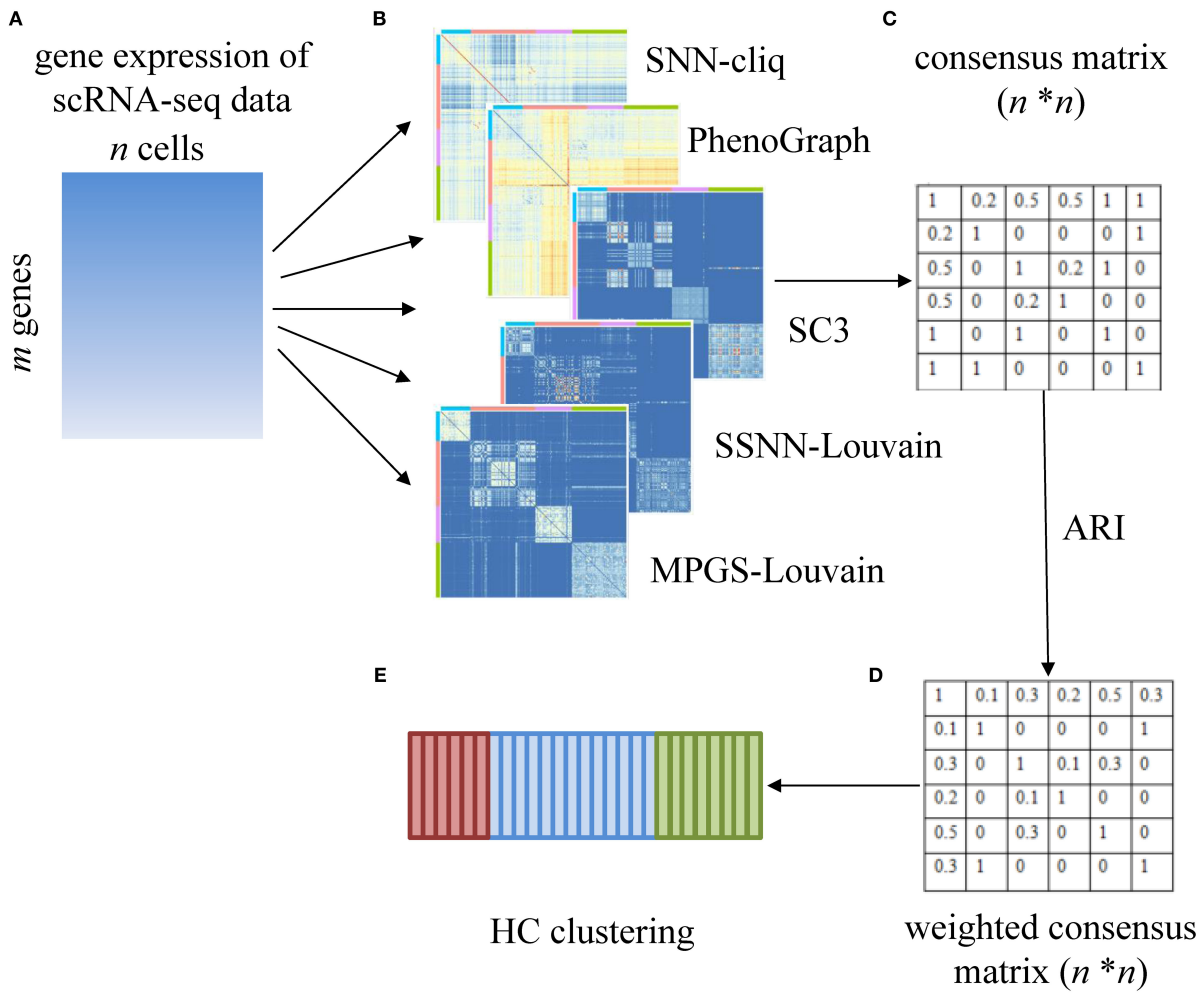
The overview of the Sc-GPE method. **(A)** The gene expression matrix is input; **(B)** five individual clustering methods are performed to generate five clustering solutions; **(C)** the original consensus matrix is constructed; **(D)** the weighted consensus matrix is produced by measuring the importance of the individual clustering methods; **(E)** HC clustering is performed.

In Sc-GPE, a gene expression matrix with *m* rows (genes) and *n* columns (cells) is the input of the five clustering methods. The five clustering results sets are achieved and ensembled into a consensus matrix with *n* rows (cells) and *n* columns (cells). Then, based on the consensus matrix, a weighted consensus matrix is constructed by measuring the importance of the individual clustering method. That is, the voting strategy in the original consensus matrix is replaced as a weighted voting strategy, and the weight is determined according to the similarity of the clustering result pairs. The overview of Sc-GPE method is shown in Figure 1.

Cells are defined as set *C* = {*c*_1_, …, *c*_*n*_}, where *n* is the number of cells. Let *k* be the number of individual clustering methods, the clustering results set is defined as *R*= {*R*^1^, …, *R*^*k*^}. So, in the *k* clustering methods, the *i*-th cell *c*_*i*_ is assigned to *k* predicted cluster labels, denoted as *R*(*c*_*i*_) = {*R*^1^(*c*_*i*_), …, *R*^*k*^(*c*_*i*_)}. The detail of Sc-GPE is described as follows.

Firstly, the original consensus matrix is constructed. The consensus matrix ***I***_*x, y*_ is calculated based on Equations (1) and (2). In Equations (1) and (2), when the cell *c*_*x*_ and cell *c*_*y*_ are assigned into the same cluster in the *l*-th method, the value of $\delta \left(R^{l}\left(c_{x}\right),R^{l}\left(c_{y}\right)\right)$ is equal to 1, otherwise is 0. The element of the consensus matrix presents the probability of cell pairs divided into the same cluster by each method. For example, when *k* is 5, the element of the consensus matrix ***I***_*x, y*_ equals the sum of $\delta \left(R^{l}\left(c_{x}\right),R^{l}\left(c_{y}\right)\right)$ in the five methods multiplying by the same weight 1/5. Because this represents the probability of the occurrence of cell pairs in the same cluster, this strategy does not need to solve the problem that each cell achieves different cluster labels from the individual clustering methods.

(1) $$\begin{array}{l} I_{x,y}=\frac{1}{k}\overset{k}{\underset{l=1}{\sum }}\delta \left(R^{l}\left(c_{x}\right),R^{l}\left(c_{y}\right)\right) \end{array}$$

(2) $$\begin{array}{l} \delta \left(X,Y\right)=\left\{ \begin{array}{c} 0,ifX\neq Y\\ 1,ifX=Y, \end{array}\right. \end{array}$$

where *c*_*x*_ and *c*_*y*_ are cell pairs in cells set *C*. *k* is the number of individual clustering methods. *R*^*l*^ is the clustering results in the *l*-th method.

Next, based on the assumption that the more consistent cluster labels predicted by all the clustering methods are more accurate, we design an importance score of the individual clustering methods. As ARI is a popular index for measuring the consensus of two clustering solutions, we use ARI to measure the importance of the individual clustering method. The importance score is defined as Equations (3) and (4). In Equations (3) and (4), ω_*l*_ denotes the importance of the *l*-th clustering method in all *k* methods. *r*_*l*_ represents the similarity between the *l*-th clustering method and other methods, which is calculated by averaging the ARI between predicted clusters in the *l*-th clustering method and the ones in each of the other methods.

(3) $$\begin{array}{l} \omega_{l}=\frac{r_{l}}{\overset{k}{\underset{j=1}{\sum }}r_{j}} \end{array}$$

(4) $$\begin{array}{l} r_{l}=\frac{1}{k-1}\overset{k}{\underset{j=1,j\neq l}{\sum }}ARI\left(R^{l},R^{j}\right), \end{array}$$

where ω_*l*_ is the importance score of the *l*-th clustering method. *r*_*l*_ is the average of ARI between predicted clusters from the *l*-th method and other methods, and *k* is the number of individual clustering methods.

Then, the weighted consensus matrix is constructed by introducing the importance score of the individual clustering method to the original consensus matrix. The weighted consensus matrix ***I***_*x, y*_' is defined as Equation (5). In Equation (5), the weighted consensus matrix ***I***_*x, y*_' multiplies the importance score ω_*l*_ of the individual clustering methods, instead of the constant 1/*k* in the original consensus matrix.

(5) $$\begin{array}{l} {I_{x,y}}^{\prime }=\overset{k}{\underset{l=1}{\sum }}\omega_{l}\times \delta \left(R^{l}\left(c_{x}\right),R^{l}\left(c_{y}\right)\right), \end{array}$$

Finally, the HC method is performed to cluster cells on the weighted consensus matrix.

### Evaluation Indices

We use two popular indices to evaluate the performance of clustering methods, including Normalized Mutual Information (NMI) (Estévez et al., 2009) and Adjusted Rand Index (ARI) (Hubert and Arabie, 1985). The two criteria are statistic-based indicators, showing the consensus of the predicted labels and the true ones in different views. NMI demonstrates the difference by calculating Mutual Information and Entropy between the two clustering solutions, with the range of values from 0 to 1. ARI presents the probability that a data pair will appear in the same cluster in the true clusters and the predicted clusters, with the range of values from −1 to 1. The higher the NMI or ARI value obtained, the better performance the method has.

(6) $$\begin{array}{l} NMI\left(P,Q\right)=2\frac{I\left(P;Q\right)}{H\left(P\right)+H\left(Q\right)}, \end{array}$$

where *I*(*P*; *Q*) is the mutual information between *P* and *Q*. H(*P*) and H(*Q*) is the entropy of *P* and *Q*, respectively.

(7) $$\begin{array}{l} ARI=\frac{\underset{i,j}{\sum }\left(\begin{array}{c} n_{ij}\\ 2 \end{array}\right)-\left[\underset{i}{\sum }\left(\begin{array}{c} a_{i}\\ 2 \end{array}\right)\underset{j}{\sum }\left(\begin{array}{c} b_{j}\\ 2 \end{array}\right)\right]/\left(\begin{array}{c} n\\ 2 \end{array}\right)}{\frac{1}{2}\left[\underset{i}{\sum }\left(\begin{array}{c} a_{i}\\ 2 \end{array}\right)+\underset{j}{\sum }\left(\begin{array}{c} b_{j}\\ 2 \end{array}\right)\right]-\left[\underset{i}{\sum }\left(\begin{array}{c} a_{i}\\ 2 \end{array}\right)\underset{j}{\sum }\left(\begin{array}{c} b_{j}\\ 2 \end{array}\right)\right]/\left(\begin{array}{c} n\\ 2 \end{array}\right)}, \end{array}$$

where *n* is the number of cells. In the contingency table resulting from the overlap between true clusters and predicted ones, *n*_*ij*_ is the element in the *i*-th row and the *j*-th column, *a*_*i*_ is the summation of the elements in the *i*-th row, and *b*_*j*_ is the summation of the elements in the *j*-th column.

### Datasets

We collected 12 published scRNA-seq datasets. Generally, they serve as gold standard datasets with true labels. They are available from Gene Expression Omnibus (GEO) and European Bioinformatics Institute (EMBL-EBI), respectively. These datasets have been normalized to various units, such as Transcripts Per Million reads (TPM), Fragments Per Kilobase of transcript per Million fragments mapped (FPKM), and Reads Per Kilobase per Million mapped reads (RPKM), etc. The details of the datasets are presented in Table 1.

**Table 1:** The detail of scRNA-seq datasets.

**Accessed ID**	**Datasets**	**Data unit**	**#Cells**	**#Genes**	**#Cell types**	**References**
GSE38495	Ramskold	RPKM	33	21042	7	Ramsköld et al., 2012
GSE57249	Biase	FPKM	49	25384	3	Biase et al., 2014
GSE36552	Yan	RPKM	90	20214	6	Yan et al., 2013
E-MTAB-3321	Goolam	RPM	124	40315	5	Goolam et al., 2016
GSE70657	Grover	RPKM	135	15158	2	Grover et al., 2016
GSE70605	Liu	RPKM	145	18855	25	Liu et al., 2016
GSE51372	Ting	RPM	187	21583	7	Ting et al., 2014
GSE85908	Yeo	TPM	214	27473	4	Song et al., 2017
E-MTAB-2805	Pollen	TPM	249	6982	11	Pollen et al., 2014
GSE45719	Deng	RPKM	259	22147	10	Deng et al., 2014
GSE52529	Trapnell	FPKM	372	35988	4	Trapnell et al., 2014
GSE67835	Darmanis	CPM	466	22085	9	Darmanis et al., 2015

## Experiments and Results

### Implementation of the Five Clustering Methods

For optimal performance, we performed the five clustering methods with the default parameters in the references. The details of the parameters are described as follows.

For SNN-cliq, the nearest neighbor parameter *k* is set to 3; the connectivity parameter of quasi-cliques *r* is set to 0.7; the threshold of the overlap of quasi-cliques *m* is set to 0.5.

For PhenoGraph, the surface marker expression data is normalized based on dividing by the maximum values. To construct the SNN graph, the nearest neighbor parameter *k* is set to 50.

For SC3, the log-transformed normalized log_2_(*x*+1) is performed.

For SSNN-Louvain and MPGS-Louvain, SIMLR is performed with the default parameters in the initial similarity measurement step. The width parameter of the Gaussian kernel function σ is set to 1.0, 1.25, 1.5, 1.75, and 2. The nearest neighbor parameter *k* is set to 10, 12, 14… 30. (σ, *k*) pair resulting in 55 Gaussian kernels. In SSNN-Louvain, to construct the structural SNN graph, the nearest neighbor parameter *k* is set to 0.1*n* (*n* is the number of nodes). In MPGS-Louvain, the path length *l* is set to 2 for high performance and low time complexity.

Furthermore, in SNN-cliq, PhenoGraph, SSNN-Louvain, and MPGS-Louvain, the number of categories can be automatically estimated by using quasi-clique partition or Louvain community detection, without a priori true categories.

### Similarity Measurement of the Individual Clustering Methods

**Figure 2:**
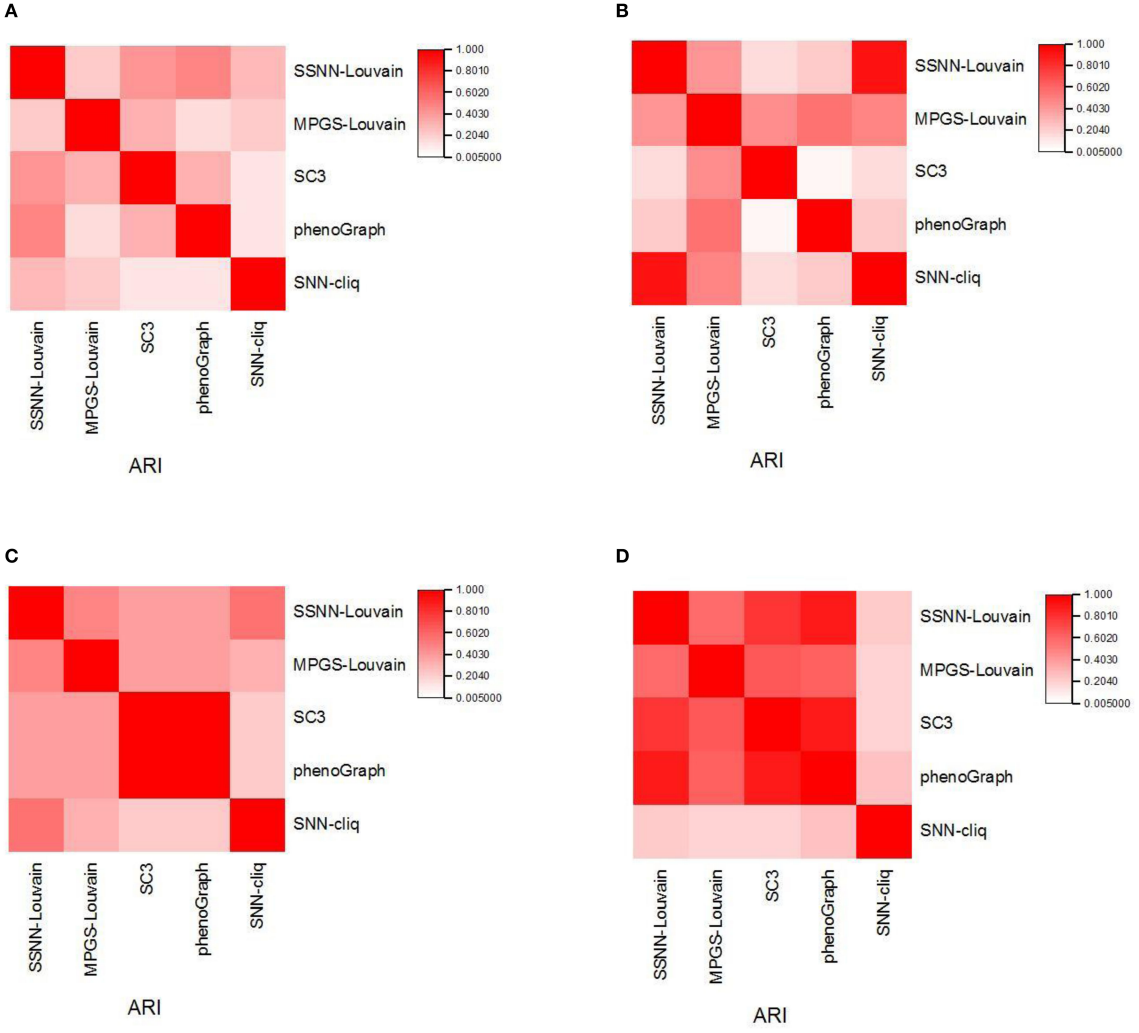
The similarity of the individual clustering methods. **(A)** Liu dataset; **(B)** Ramskold dataset; **(C)** Yan dataset; **(D)** Yeo dataset.

To analyze the difference of predicted results between the individual clustering methods, we calculate the ARI between the different clustering results and provide the consensus matrix heatmap. We select four scRNA-seq datasets: Ramskold, Yan, Yeo, and Liu, in which the Ramskold dataset is easy to partition while the Liu dataset is hard to cluster. The first three datasets have a smaller number of true categories from four to seven, and the latter dataset has the true categories 25. The heatmaps are shown in Figure 2.

From Figure 2, it is observed that some faint similarity exists among the solutions of the individual clustering methods, which is consistent with the results from Yang et al. (2019). In different datasets, the similarities between the results of the individual clustering methods vary. For example, SSNN-Louvain shows relatively high similarity with SC3 and PhenoGraph on the Liu dataset. MPGS-Louvain shows a higher similarity than other clustering methods to the Ramskold dataset. SC3 is observed in the high similar to PhenoGraph on the Yan dataset. SNN-cliq shows a low similarity with other methods on the Yeo dataset. The difference between SC3 and PhenoGraph varies greatly in different datasets. The similarity between SC3 and PhenoGraph is close to one on the Yan and Yeo datasets, but the opposite results are achieved on the Liu and Ramskold datasets.

Furthermore, we can observe big differences between SNN-cliq and SC3, PhenoGraph on the four datasets. Therefore, we can find that different clustering methods would capture information about scRNA-seq data from different perspectives.

### Comparisons With the Individual Clustering Methods and SAME-Clustering

**Figure 3:**
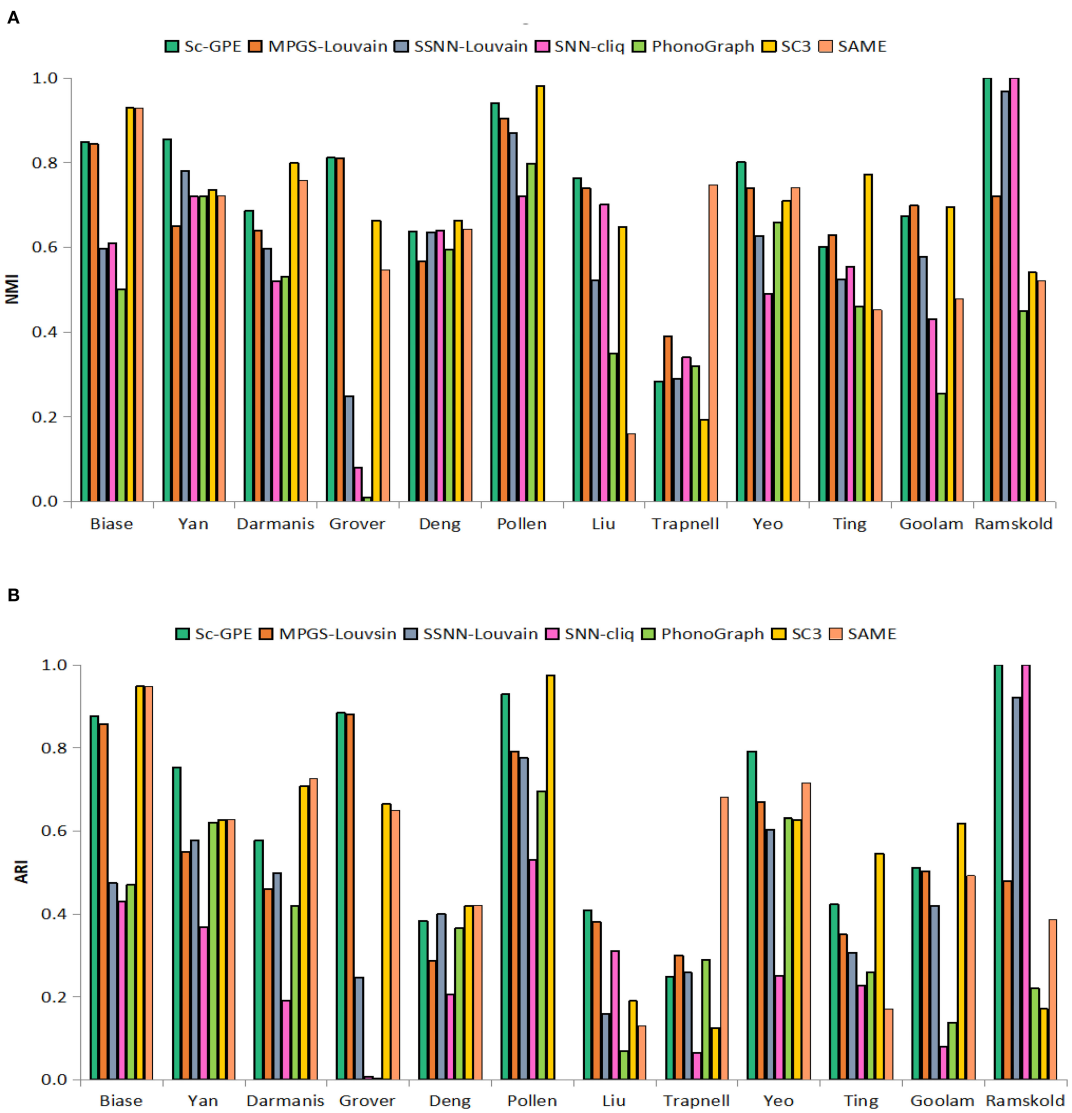
The performance of Sc-GPE, MPGS-Louvain, SSNN-Louvain, SSNN-cliq, PhenoGraph, and SC3. **(A)** NMI; **(B)** ARI.

To test the performance of our proposed Sc-GPE method, we compare it with both the five clustering methods and the state-of-the-art clustering ensemble algorithm SAME-clustering on 12 scRNA-seq datasets in terms of NMI and ARI. The results are shown in Figure 3. SAME-Clustering achieves the NA value of NMI and ARI on the Pollen dataset, because the clustering member Seurat in SAME-Clustering failed to run on this dataset.

From the experimental results, Sc-GPE achieves the highest average of NMI and ARI in all methods. Sc-GPE outperforms the six methods on five scRNA-seq datasets: Yan, Grover, Liu, Yeo, and Ramskold, while SC3 achieves the best performance on five scRNA-seq datasets: Biase, Deng, Pollen, Ting, and Goolam. The averages of NMI and ARI obtained by Sc-GPE are 6.92 and 17.79% higher than those of SC3, respectively. SAME-Clustering works best on three datasets: Biase, Darmanis, and Trapnell. The averages of NMI and ARI obtained by Sc-GPE are 21.84 and 20.19% higher than those of SAME-clustering, respectively. A large difference in clustering performance can be observed on the Grover, Liu, and Goolam datasets. The results show that Sc-GPE performs well and outperforms other methods.

Moreover, we compare the number of clusters in the seven methods, shown in Table 2. It can be observed that the number of predicted clusters has an obvious influence on the clustering solutions. For example, the clustering number of SNN-cliq and PhonoGraph is quite different from that of other methods, which is in consensus with their relatively poor performance on most datasets. SNN-cliq achieves the clustering numbers commonly more than the true categories except for the pollen dataset, PhonoGraph is just the opposite.

**Table 2:** The comparison of the number of clusters from seven methods.

**Datasets**	**Sc-GPE**	**MPGS-Louvain**	**SSNN-Louvain**	**SNN-cliq**	**PhonoGraph**	**SC3**	**SAME-clustering**
Ramskold	7	3	8	7	2	2	2
Biase	3	3	4	6	2	3	3
Yan	6	6	8	18	3	3	3
Goolam	5	5	6	25	4	2	3
Grover	2	2	3	12	3	3	2
Liu	25	15	7	26	3	6	4
Ting	7	8	7	21	5	11	4
Yeo	4	5	3	28	3	5	3
Pollen	11	11	7	9	7	11	NA^*^
Deng	10	10	7	43	6	6	5
Trapnell	4	5	6	56	6	10	4
Darmanis	9	8	5	38	6	12	5

* *SAME-Clustering method achieves NA on the Pollen dataset for that the clustering member Seurat in SAME-Clustering failed to run on this dataset*.

**Figure 4:**
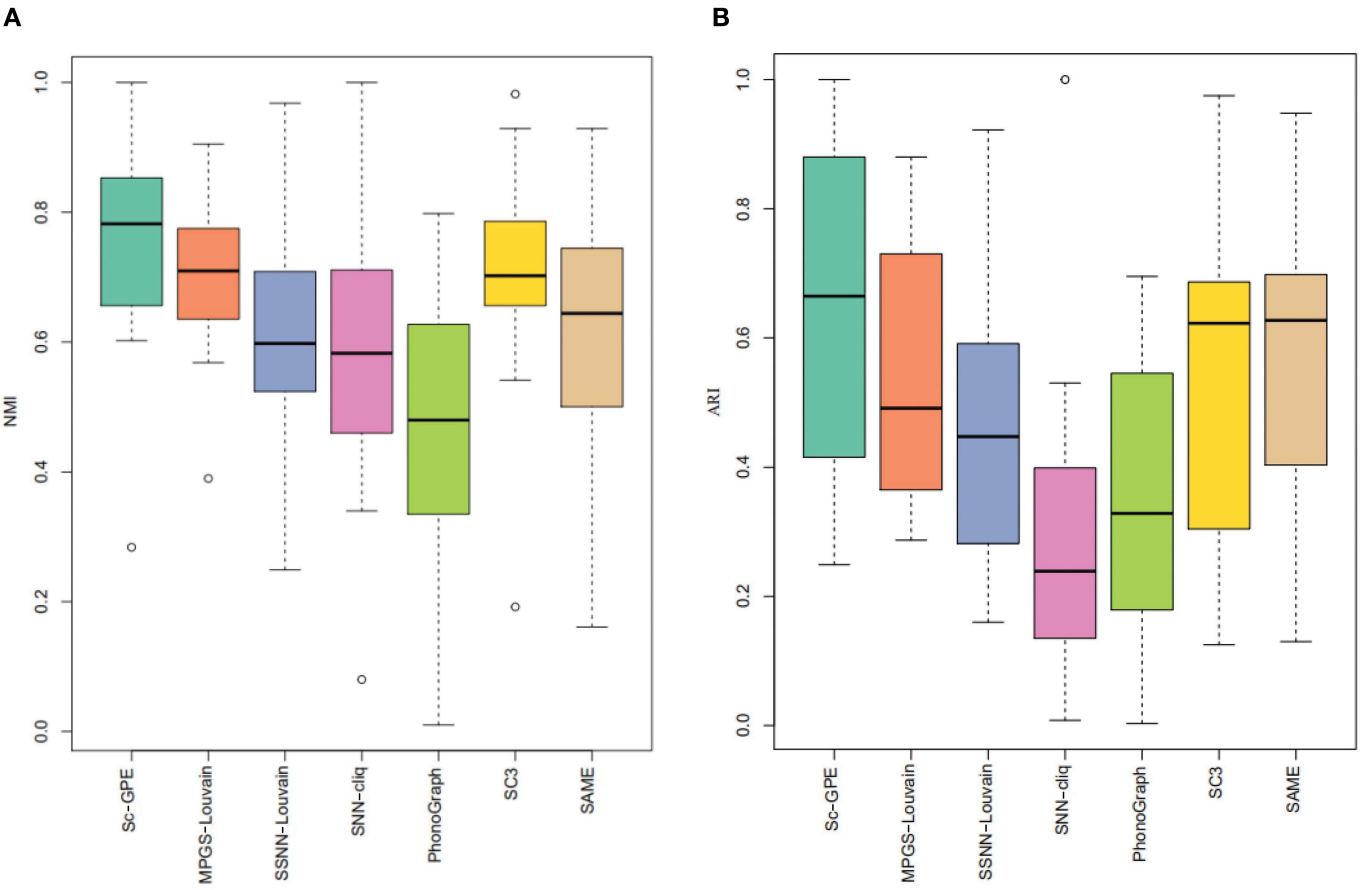
The box plot of performance for the seven methods. **(A)** NMI; **(B)** ARI.

To further demonstrate the performance of Sc-GPE, we provide a box plot of the seven methods for 12 datasets, measured by NMI and ARI, shown in Figure 4. The box plot clearly shows that Sc-GPE outperforms the other six methods. The worse ARI value of 0.249 in Sc-GPE is from the Trapnell dataset, where some cells are misallocated resulting from two poor clustering solutions. SNN-cliq achieves the worst results in terms of ARI, and PhenoGraph performs worst on the NMI.

## Conclusions

Currently, various single-cell clustering algorithms have been proposed with the advantage of accurately representing cell heterogeneity. However, there is a problem that the predicted cluster results from different clustering methods are quite different, which would limit the generalization capabilities. Combining the information from different cluster results would be a good resolution to improve the performance of clustering. So, we propose a novel cluster ensemble method Sc-GPE, which integrating five clustering methods: SNN-cliq, PhenoGraph, SSNN-Louvain, MPGS-Louvain, and SC3.

In Sc-GPE, a consensus matrix-based ensemble model is performed. It is a good statistics approach that can solve the problem of the different cluster labels generated in the individual clustering methods making it difficult to determine the correspondence cluster labels across all methods, which usually exists in the hypergraph-based cluster ensemble method. Furthermore, a weighted strategy is designed to measure the importance of individual clustering methods according to the similarity with other methods. A weighted consensus matrix is constructed based on the weighted strategy, which can distinguish the role of the individual clustering methods.

Sc-GPE provides close-to-the-best clustering solutions by combing the clustering methods that perform various similarity measurements and graph partitioning algorithms. The experimental results from twelve scRNA-seq datasets show that Sc-GPE outperforms the five individual clustering methods and state-of-the-art SAME-clustering method. However, the relatively small number of individual clustering methods may provide insufficient information and limit the performance of the Sc-GPE, and how to choose more optimal individual clustering methods should be researched in future work.

## Data Availability Statement

The datasets analyzed in this work are available in the following repositories: GEO: https://xenabrowser.net/datapages/; EMBL-EBI: https://www.ebi.ac.uk/ and details of the datasets can be found in Table 1.

## Author Contributions

XZ and JW: conceptualization and design. XZ and H-DL: writing. H-DL and MX: data acquisition. XZ and JL: methodology. All authors: contributed to the article and approved the submitted version.

## Conflict of Interest

The authors declare that the research was conducted in the absence of any commercial or financial relationships that could be construed as a potential conflict of interest.
